# Potential bias in observational PCSK9 inhibitor study

**DOI:** 10.1093/ehjcvp/pvaf041

**Published:** 2025-05-22

**Authors:** Joseph Edgar Blais, Angel Y S Wong

**Affiliations:** Department of Pharmacology and Pharmacy, Centre for Safe Medication Practice and Research, LKS Faculty of Medicine, The University of Hong Kong, L2-54 Laboratory Block, 21 Sassoon Road, Pokfulam, Hong Kong SAR, China; Faculty of Epidemiology and Population Health, London School of Hygiene and Tropical Medicine, Keppel Street, London WC1E 7HT, UK

We read with interest the study by Huang et al.^1^ published in the *European Heart Journal—Cardiovascular Pharmacotherapy*.^1^ Their study attempts to provide insights into the effectiveness of proprotein convertase subtilisin/kexin type 9 (PCSK9) inhibitors against all-cause mortality and hospitalization-related outcomes. It is also an example of how researchers’ decisions about the design of their pharmacoepidemiological study can result in biased estimates. Several different biases can occur in both the design and analysis of observational studies that use real-world data. These biases often occur because of the growing availability of longitudinal electronic databases which permit investigators to look both forwards and backwards in calendar time.^2,3^ Looking into the future and making decisions about an individual’s eligibility to be included in a study is simply not possible in a randomized controlled trial or cohort study with primary data collection. Analysis decisions, such as identifying treatment groups one before the other or excluding people from the analysis on the basis of the number of visits, their diagnoses, or their treatment during follow-up can cause selection bias.^4,5^

In the study conducted by Huang et al.^1^ the authors defined the index date (time zero) as initiation of PCSK9 inhibitors and statins respectively. In the PCSK9 inhibitor treatment strategy, individuals were allowed to be prescribed a statin before the index date. Therefore, they were not incident (new) users of lipid lowering drugs, which means some individuals receiving the PCSK9 inhibitor treatment strategy would have a survival advantage over the statin treatment strategy. Moreover, as switchers were excluded from the analysis (i.e. for the PCSK9 inhibitor treatment strategy, individuals prescribed a statin ‘on or after the index date’) were excluded instead of censoring the follow-up time at treatment switching. This excludes the person-time for individuals in the statin treatment strategy that were subsequently prescribed a PCSK9 inhibitor and the person-time of individuals in the PCSK9 inhibitor treatment strategy that were subsequently prescribed a statin. In a randomized clinical trial, time zero is the date when participant eligibility is assessed, treatment strategies are assigned, and follow-up starts.^3^ As described by Hernán and colleagues, misalignment of these three decisions frequently occurs in observational studies and produces selection bias.^3^ The study design selected by Huang et al.^1^ suggests misalignment of time zero (specifically target trial emulation failure example #4 described by Hernán) and is an example of classical immortal time bias that could bias the results in favour of PCSK9 inhibitors.^5^

Mohyuddin and Prasad also describe how bias can be identified by inspecting the survival plots to understand how quickly an intervention exerts an effect on the outcome under study.^6^ Inspecting *Figure 2* in Huang et al.^1^ shows a clear separation of the cumulative incidence of all-cause mortality immediately after 30 days of follow-up. The speed of this effect against all-cause mortality seems biologically improbable given that the landmark ODYSSEY OUTCOMES and FOURIER studies showed that the survival curves for all-cause mortality began to separate after 12 months of treatment.^7,8^ Lastly, defining the start of follow-up at 30 days after the index date causes selection bias in the hazard ratio because of depletion of susceptible individuals.^9^ The susceptibility of individuals to all-cause mortality does not remain constant over time as shown in Supplementary material online, *Table S9* of the original paper. A higher risk of mortality might be expected, for example, within one month after a patient has a myocardial infarction who then goes on to initiate a statin at hospital discharge. This depletion of susceptibles may also contribute to the rapid separation of the survival curves at the start of follow-up.

How can these flaws in real-world data analysis be avoided? Clinical investigators who intend to use complex real-world data need to include team members with expertise in observational epidemiology and who are ideally experienced with the health system and data source used in the study. Using the target trial framework can help avoid common errors in study design.^3^ Furthermore, the questions listed in Lévesque et al.^5^ can also be used by researchers to avoid inducing immortal time bias.^5^ Study design diagrams and other visualisations have been promoted to explicitly describe the order of application of time windows for each temporal anchor and patient flow in real-world studies.^10^

An increase in the accessibility of electronic health records such as the TriNetX database should come with a commensurate increase in the responsibilities of authors, journal editors, and peer reviewers to ensure the application of appropriate methodologies to mitigate bias in real-world studies of cardiovascular pharmacotherapy. Researchers in pharmacoepidemiology are in danger of losing trust and credibility if we fail to appropriately respond to these methodological challenges when analyzing real-world data.
